# Exploring the Mechanical and Thermal Impact of Natural Fillers on Thermoplastic Polyurethane and Styrene–Butadiene Rubber Footwear Sole Materials

**DOI:** 10.3390/polym16223201

**Published:** 2024-11-18

**Authors:** Víctor M. Serrano-Martínez, Henoc Pérez-Aguilar, María Pilar Carbonell-Blasco, Cristina Llobell-Andrés, Francisca Aran-Ais, Avelina García-García, Elena Orgilés-Calpena

**Affiliations:** 1 Footwear Technology Centre, Campo Alto Campground, 03600 Alicante, Spain; 2 MCMA Group, Department of Inorganic Chemistry and Institute of Materials, University of Alicante, Carretera de Sant Vicent del Raspeig, s/n, 03609 Sant Vicent del Raspeig, Spain

**Keywords:** rice straw, cellulose, SBR soles, TPU soles, mechanical properties, experimental design, thermal properties, revalorization, footwear industry

## Abstract

The increasing concern for sustainability in the footwear industry has spurred the exploration of eco-friendly alternatives for materials commonly used in sole manufacturing. This study examined the effect of incorporating rice straw and cellulose as fillers into soles made from either styrene–butadiene rubber (SBR) or thermoplastic polyurethane (TPU). Both fillers were used as a substitute in mass percentages ranging from 5 to 20% in the original SBR and TPU formulas, and their impact on mechanical properties such as abrasion and tear resistance, as well as thermal properties, was thoroughly evaluated. The results demonstrated that the inclusion of fillers affects the overall performance of the soles, with the optimal balance of mechanical and thermal properties observed at a 10% filler content. At this level, improvements in durability were achieved without significantly compromising flexibility or abrasion resistance. Thermal analysis revealed increased thermal stability at moderate filler contents. This research not only offers a sustainable alternative to traditional materials but also enhances sole performance by improving the composition. Furthermore, this study paves the way for future research on the feasibility of incorporating eco-friendly materials into other consumer product applications, highlighting a commitment to innovation and sustainability in product design.

## 1. Introduction

Environmental sustainability is crucial in the manufacturing industry, particularly in the footwear sector [1]. Consumers are increasingly demanding sustainable practices, leading companies to explore eco-friendly materials and production methods [2]. Conventional sole materials like styrene–butadiene rubber (SBR) and thermoplastic polyurethane (TPU) pose environmental challenges due to their production processes and disposal issues. These materials are derived from non-renewable petroleum sources, and their production involves energy-intensive processes that emit considerable amounts of greenhouse gases. Moreover, at the end of their life cycle, these materials are non-biodegradable, leading to accumulation in landfills and contributing to pollution and resource depletion [3]. The fashion industry, including the footwear sector, is a significant contributor to global climate emissions, emphasizing the need for sustainable alternatives. Biodegradable shoe development is gaining traction as a solution to enhance sustainability in footwear, aligning with consumer preferences for environmentally friendly products.

As such, previous studies in the footwear sector have increasingly focused on the use of alternative materials, including recycled and biodegradable options, to address environmental concerns and improve sustainability [4,5,6]. For instance, research by Bianchi et al. explored the use of recycled Ethylene Vinyl Acetate (EVA) in outsole manufacturing, demonstrating that the approach offered significant environmental and cost benefits despite the mechanical properties of recycled EVA being lower than virgin EVA, suggesting a viable path towards a zero-waste production system in the footwear industry [7]. Similarly, the work conducted by Bashpa et al. on reutilizing shoe sole scrap from thermoset polyurethane (PU) as a reinforcing filler in natural rubber composites showed improved tensile strength and abrasion resistance at certain filler loadings, indicating the potential for high-performance, low-cost footwear applications, albeit with some limitations in mechanical properties at higher filler loadings [8]. The exploration of biodegradable materials for shoe manufacturing also reflects a growing consumer demand for sustainable products. A study on consumer acceptance of biodegradable shoes highlighted the importance of aesthetics alongside sustainability, suggesting that transparent communication of brand values could enhance market acceptance of environmentally friendly footwear options [3]. However, the challenge remains in balancing the mechanical and aesthetic properties of sustainable materials to meet consumer expectations. These studies collectively underscore the advances in using recycled, biodegradable, and naturally sourced materials in the footwear sector, pointing towards improved sustainability and reduced environmental impact. However, they also reveal limitations related to the mechanical properties and commercial viability of alternative materials, indicating areas for further research and development.

Previous studies have demonstrated the potential of rice straw and cellulose in various applications, yet there remains a significant gap in research specifically targeting their use in SBR and TPU soles. Research on the mechanical reinforcement of high-density polyethylene with rice-straw-derived nanofibres has shown improvements in strength, hardness, and wear resistance [9]. Furthermore, the versatility and mechanical benefits of rice straw have also been evident in its successful use in concrete and wall panels [10] and in the development of bioplastics [11]. Additionally, modifications of rice bran oil for enhanced lubricity and oxidation stability have suggested potential benefits for improving the wear resistance and durability in composites [12,13,14]. On the other hand, cellulose-based composites have gained significant attention due to their environmental benefits and versatility in various applications. The use of cellulose as reinforcement in polymer composites has shown improvements in mechanical properties, such as tensile strength and Young’s modulus [15]. Reviews have highlighted its renewability, low cost, and positive environmental impact, with composites demonstrating enhanced strength and stiffness [16,17]. However, challenges remain in optimizing cellulose material composites for industrial-scale production and application [18]. Despite these promising findings, neither rice straw nor cellulose has been directly applied to the development of footwear soles, indicating an area ripe for further exploration and innovation.

Building on these observations, this research aimed to fill the existing gap by providing a detailed analysis of how the incorporation of two natural fillers, rice straw and cellulose, could fundamentally alter the mechanical and thermal properties of TPU and SBR soles. In this context, it was anticipated that adding rice straw or cellulose would enhance the mechanical properties of the soles, given their natural fibrous nature and mechanical strength, which contribute positively to the overall performance of composite materials [19,20].

For the experimental work, the cellulose used was extracted from rice straw as reported in a previous article [21], employing a steam explosion process to enhance its suitability for composite materials. This method was chosen for its ability to effectively break down the lignocellulosic matrix, increasing surface area and porosity, and preserving the fibrous integrity and mechanical properties of the cellulose. The subsequent experimental phase involved the preparation of composite soles samples by varying the substitution of the percentages of rice straw and cellulose, followed by rigorous testing of their mechanical properties, including abrasion resistance, tensile strength, elongation at break, and tear resistance, to identify optimal formulations, as well as thermal properties based on thermogravimetric analysis (TGA) and differential scanning calorimetry (DSC). This approach is supported by the successful application of similar materials in other contexts [22,23,24], suggesting a promising avenue for enhancing footwear material technology.

Moreover, the outcomes of this study hold significant implications for both the footwear industry and broader sustainability initiatives. By demonstrating the feasibility of using rice straw and cellulose in sole manufacturing, the authors provide a pathway for reducing reliance on non-renewable resources and mitigating environmental impacts associated with conventional sole materials. This aligns with global efforts to promote sustainable production practices and supports the transition towards a circular economy model, while balancing product performance with environmental responsibility. Overall, this study contributes to advancing sustainable materials in the footwear industry and underscores the potential for meaningful contributions to both environmental conservation and industry innovation.

## 2. Materials and Methods

In this study, laboratory-scale samples of SBR and TPU were prepared by incorporating rice straw and cellulose as fillers in varying amounts (0–20% by mass). The filler content replaced an equivalent proportion of the original formulation components to maintain a consistent 100% composition. After preparation, the samples were analysed to observe how the inclusion of these natural fillers impacts the performance of the materials, with a focus on mechanical and thermal properties.

### 2.1. Materials

Rice straw was sourced from the Albufera of Valencia, a renowned wetland and rice-growing area in Spain. This material was supplied by Unió Llauradors i Ramaders (Valencia, Spain), a prominent organization representing agricultural professionals in the region, known for its commitment to sustainable farming practices. The rice straw, a by-product of these fields, is commonly available post-harvest. On the other hand, the cellulose used in this study was extracted through an optimized process developed by INESCOP [19], which uses the same rice straw. This process involves a steam explosion, where the material is subjected to high temperatures and pressures followed by rapid decompression, breaking down the lignocellulosic structure and facilitating selective cellulose extraction. Additional steps included alkaline pre-treatment and hydrogen peroxide bleaching to obtain cellulose with high purity. For this study, both rice straw and cellulose were milled using a Retsch Emax ball mill (Haan, Germany), a high-energy ball mill with 2600 W of power, which allowed the particle size to be reduced to less than 1 mm. This process ensures efficient and uniform milling, suitable for preparing the fillers for subsequent use in composite materials.

The other materials used in the formulations studied included styrene–butadiene rubber (SBR 1502), silica (99.8% purity, particle size <250 µm, specific surface area of 180 m^2^/g), TDAE oil (Extensoil 1996, cinematic viscosity 389.9 mm^2^/s at 40 °C, aniline point 80 °C), zinc oxide (99.8% purity, particle size <45 µm, specific surface area 4–6 m^2^/g), stearic acid (CYPTEC AE-01-V, 65% vegetable-derived), polyethylene glycol (PEG 4000), benzothiazyl disulphide (MBTS, 94% purity), tetramethylthiuram disulphide (TMTD, 95% purity), N′,N-diphenylguanidine (DPG, 99.9% purity), and sulphur (99% purity), all sourced from MCE Mezclas caucho S.A.U. (Elche, Spain). The TDAE oil was chosen for its low aromatic content, making it a suitable extender oil that enhances the rubber’s flexibility and compatibility with bio-based fillers. The thermoplastic polyurethane (TPU-Wantane) in granule form, also sourced from MCE Mezclas caucho S.A.U., was used as the matrix for some formulations.

### 2.2. Preparation of Bio-Based SBR with Rice Straw and Cellulose

**Figure 1:**
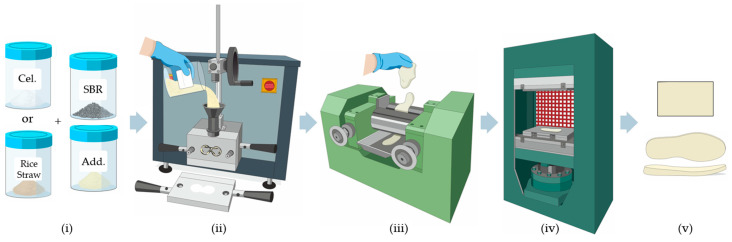
Scheme of mixing (**i**,**ii**), calendaring (**iii**), and pressing (**iv**) of different SBR samples (**v**).

Vulcanized SBR mixtures were prepared, with two types of fillers used to substitute the SBR formula: rice straw and cellulose in a concentration range of 0–20% by mass. The compounds used (i) in the formulation are detailed in Table 1. The various mixtures were prepared in a Haake Polylab QC mixer (Thermo Fisher Scientific S.L.U., Madrid, Spain) using Banbury rotors (ii), with a net chamber volume of 78 cm^3^, operating at 30 rpm with a mixing time of 10 min and a temperature of 160 °C [25,26]. Figure 1 illustrates the complete process followed for the fabrication of the analysed samples.

The quantities of the compounds in Table 1 were adjusted proportionally to ensure that, when the filler (rice straw or cellulose) was added at concentrations ranging from 0 to 20% by mass, the total composition always summed to 100%, thus proportionally reducing the rest of the compounds. This approach was used to maintain consistency across all formulations.

After obtaining the mixture in the Haake Polylab QC, it was calendered to obtain preforms using a Gumix GX-2403C (Farrel Spain S.L., Barcelona, Spain) roller mixer (iii). The calendering process was carried out in ‘calender mode’ at a fixed speed of 16 rpm, without applying shearing forces and without heating the rollers, ensuring a homogeneous mixture of the compounds before the vulcanization step.

To define the press conditions for obtaining the final SBR from the preforms, a rheometric study was conducted using an MDR 2000 rheometer (Alpha Technologies, distributed by Intmatic S.L., Barcelona, Spain) at 160 °C for 20 min, with an oscillation amplitude and frequency of 0.5° and 1.7 Hz, respectively [27].

Finally, to perform various physical characterization tests, the preforms were vulcanized in a GUIX GX-1105 (Guix Machines, S.L., Barcelona, Spain) hydraulic press (iv) into 100 × 50 × 2 mm sheets of rubber compounds, at 160 °C, 100 bar pressure, and different residence times defined by the rheometric study.

Section 3 presents and analyses the obtained results, including vulcanization curves, maximum and minimum elastic pairs, vulcanization times, and SBR sheets with different fillers and amounts added.

### 2.3. Preparation of Bio-Based TPU with Rice Straw and Cellulose

**Figure 2:**
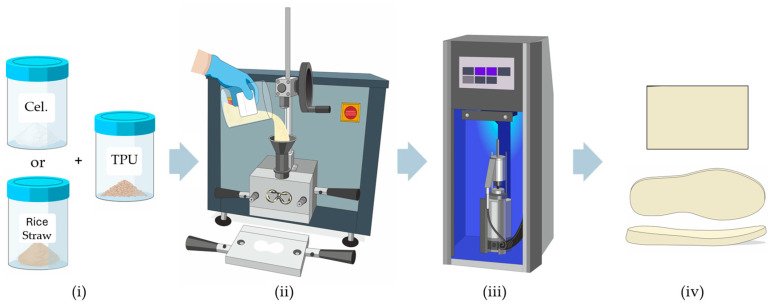
Scheme of mixing (**i**,**ii**) and injection (**iii**) for the different TPU samples (**iv**).

TPU mixtures were prepared with two types of fillers used to substitute the TPU formula: rice straw and cellulose in a concentration range of 0–20% by mass. The base formulation and mixing details are presented in Table 2 for both rice straw and cellulose. As with the SBR mixtures, the filler content was adjusted proportionally to ensure that the total composition always summed to 100%. The mixtures were prepared in a Haake Polylab QC mixer using Banbury rotors (ii), with a net chamber volume of 78 cm^3^, operating at 30 rpm with a mixing time of 10 min and a temperature of 160 °C. Figure 2 illustrates the complete process followed for the fabrication of the analysed samples.

For thermoplastic compounds, the TPU was manually granulated after mixing and moulded into 90 × 22 × 2 mm plates by injection, using a Haake Minijet Pro injector (iii), with an injection capacity of approximately 10 cm^3^ of material and under conditions of 350 bar pressure, 200 °C cylinder temperature, and 100 °C mould temperature [28,29]. Section 3 shows the TPU sheets with different fillers and amounts added.

### 2.4. Experimental Techniques

The characterization of shoe soles and test specimens incorporating rice straw and cellulose as fillers in the formulation of both SBR and TPU in different percentages was carried out using the following experimental techniques.

#### 2.4.1. Hardness

The hardness of the shoe soles and test specimens was measured using a Shore A durometer (Bareiss Prüfgerätebau GmbH., model 5019, Oberdischingen, Germany) according to the ISO 48-4:2018 standard [30]. The measurement was taken at 3 s with a support, utilizing a truncated cone-shaped needle (Shore A). The degree of penetration of the needle into the material was recorded, where greater penetration indicated lower hardness. Each test was performed in triplicate to ensure accuracy.

#### 2.4.2. Density

Density was determined in g/cm^3^ according to the UNE-ISO 2781:2015, method A [31]. Using a densimeter (A&CN Scientific Inc., Toronto, ON, Canada), the mass of a sample was measured in air and then in water. The difference in weights corresponds to the volume of water displaced, thus determining the volume of the sample. The density was calculated as the ratio of the mass in air to the volume.

#### 2.4.3. Abrasion Resistance

Abrasion resistance was tested according to UNE-EN 12770:2000 using an abrasimeter (Muver, Francisco Muñoz Irles C.B., Petrer, Spain) [32]. Cylindrical samples, 16 mm in diameter and at least 6 mm thick, were moved 40 m over a controlled abrasive cloth under a force of 10 N. The loss of mass or volume was measured and is expressed in mg or mm^3^ of material loss, respectively, with the density required for unit conversion to mm^3^. Each test was performed in triplicate to ensure accuracy.

#### 2.4.4. Tensile Strength and Elongation at Break

Tensile strength and elongation at break were assessed following UNE-EN 12803:2001/AC:2002, using a universal testing machine (Instron Ltd., model 3306, Norwood, MA, USA) [33]. Dogbone-shaped specimens, which were halter type 2, S2, were stretched until rupture. Tensile strength (MPa) was calculated by dividing the breaking force (N) by the cross-sectional area (mm^2^). On the other hand, elongation at break (%) was determined by the increased distance between two reference marks on the narrow part of the specimen at the moment of rupture. Each test was performed in triplicate to ensure accuracy.

#### 2.4.5. Tear Resistance

Tear resistance was tested according to UNE-EN 12771:2000 using a universal testing machine (Instron Ltd., model 3306, Norwood, MA, USA) [34]. Pants-shaped specimens were used to measure the force necessary to completely tear the sample, expressed in N/mm. Each test was performed in triplicate to ensure accuracy.

#### 2.4.6. Differential Scanning Calorimetry (DSC)

Differential scanning calorimetry was employed to determine the thermal properties with a DSC 823^e^ STARe Systems calorimeter (Mettler-Toledo AG, Schwerzenbach, Switzerland). The experiments were conducted in an inert nitrogen atmosphere (flow rate = 80 mL·min^−1^) at a heating or cooling rate of 10 °C/min. Approximately 5–10 mg of the sample was weighed into ME-26763 Al crucibles with a 40 µL capacity, taring the base of the crucible prior to measurement. After weighing the sample, a small hole was made in the lid of the crucible using a fine, needle-like laboratory tool to ensure controlled gas flow. The lid was then sealed onto the crucible using a hermetic sealer, ensuring a tight fit. The assembled crucible was then placed into the differential scanning calorimeter for analysis. The analysis varied depending on the material characterized, although both were composed of two sequential runs each: (i) initial heating from −120 °C to 150 °C, with (ii) a repetition of the same sequence in the case of SBR; and (i) initial heating from −100 °C to 250 °C, with (ii) a repetition of the same sequence in the case of TPU [35].

#### 2.4.7. Thermogravimetric Analysis (TGA)

The thermal stability was evaluated using a TGA 2 STARe System thermal balance (Mettler-Toledo GmbH., Barcelona, Spain), equipped with STARe v16.4 software from Mettler-Toledo, Switzerland. A sample size of approximately 7 to 10 mg of the studied compound was cut from the bulk material and placed into a 70 µL alumina crucible. The samples were then heated from 30 to 600 °C at a rate of 10 °C/min under an inert nitrogen atmosphere, with a nitrogen flow rate of 30 mL/min [36].

#### 2.4.8. Statistical Analysis

The design of experiments (DoE) for this study involved a fully randomized, crossed factorial design, where all materials were tested with all fillers at various percentages. This approach allowed for a comprehensive analysis of the effects of different factors on the measured properties of the test specimens.

The data obtained were analysed using a multifactorial analysis of variance (ANOVA). An ANOVA is a statistical technique used to determine whether significant differences exist between groups or treatments for a variable of interest. It assesses within-group variability to determine if the observed differences are greater than what would be expected by chance [37]. For this statistical analysis, STATGRAPHICS Centurion v18 software was used, with a confidence level of 95% and a significance threshold of *p* < 0.05.

In this analysis, a multifactorial ANOVA was utilized to study the influence of the selected factors (types of materials, fillers, and their percentages) on the dependent variables (hardness, density, abrasion resistance, tensile strength, elongation at break, and tear resistance). The software automatically calculated the ANOVA statistics to determine whether there were significant differences between the means of the groups [38].

The F-ratio from the ANOVA table, along with the *p*-value, indicated the probability that the observed results were due to chance rather than the effects of the factors. A predefined overall significance level of α = 0.05 was used, meaning that a *p*-value less than 0.05 was considered statistically significant. This analysis allowed for the identification of significant factors and combinations of factors that influenced the dependent variables, providing a robust understanding of how the different formulations affected the performance characteristics of the test specimens [39].

## 3. Results and Discussion

To evaluate the impact of incorporating different fillers (cellulose and rice straw) into various polymeric materials (TPU and SBR), the mechanical properties of the resulting materials were assessed and compared with the reference materials without cellulose and rice straw fillers. An experimental design was conducted to evaluate the effect of replacing part of the TPU and SBR formulation with different percentages of fillers. The fillers used were cellulose and rice straw in concentrations ranging from 0% (initial formulation sample) to 5%, 10%, 15%, and 20%, expressed consistently as mass percentages throughout this study. The measured properties included abrasion resistance, tensile strength, elongation, and tear resistance. First, a detailed analysis of the results obtained for the preparation of bio-based SBR and TPU are presented below, followed by their mechanical characterization.

### 3.1. Preparation of Bio-Based SBR with Rice Straw and Cellulose

**Figure 3:**
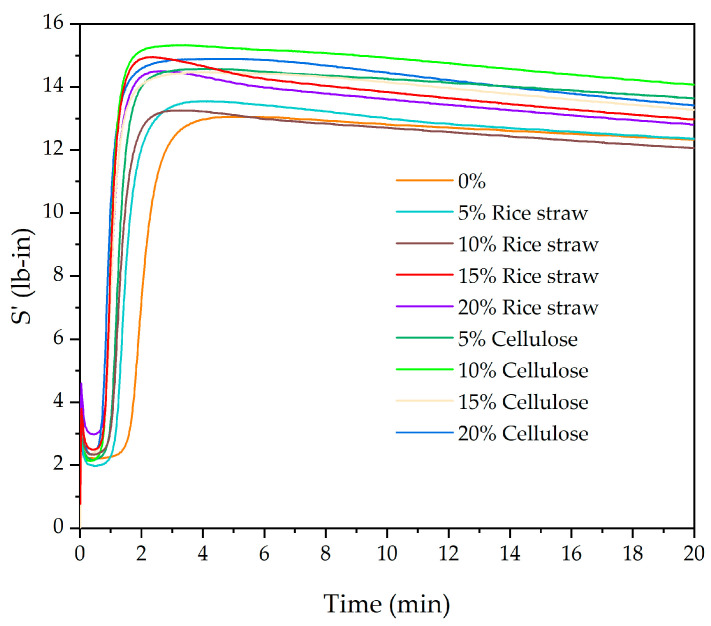
Vulcanization curves obtained for SBR-based materials with rice straw and cellulose fillers, ranging from 0 to 20%.

The vulcanization curves obtained using the rheometer are shown in Figure 3 for both rice straw and cellulose. From these curves, the results of the maximum (S’Maximum) and minimum (S’Minimum) elastic pairs, as well as the vulcanization times, have been extracted and are summarized in Table 3 and Table 4 for rice straw and cellulose, respectively.

The rheometric study shows an increase in the values of S’Maximum and S’Minimum in both cases, reflecting the higher stiffness and strength of the vulcanized compound. This reinforcement occurs as the filler particles limit the mobility of polymer chains, forming a more rigid network within the SBR matrix [40]. Additionally, a reduction in scorch times (ts 0.5 and ts 2) with the increase in the filler is observed, suggesting a faster vulcanization, which is advantageous from an industrial perspective as it reduces processing times. The presence of fillers, particularly cellulose, may act as nucleation sites, promoting cross-linking and contributing to this faster curing [41]. These data, including the optimum curing time (tc90) and scorch time, are crucial for understanding the behaviour of SBR compounds during the vulcanization process. Specifically, tc90 indicates the time required to reach 90% of total vulcanization, which is critical for establishing the optimal pressing time in sole manufacturing. Precise control of these times ensures that the compounds acquire the desired mechanical properties without compromising their structural integrity [42,43].

As indicated in Section 2, in order to be able to analyse the physical properties of the different compositions, several sheets of the rubber compounds were vulcanized, for which the residence times indicated in Table 5 were used.

The pressing times reflect the influence of rice straw and cellulose fillers on the curing process of SBR. A clear reduction in pressing time is observed as the filler content increases. For instance, at 10% cellulose, the pressing time has already decreased to 2.5 min, compared to 4.0 min for the unfilled compound. This reduction can be attributed to the enhanced nucleation effect of the fillers, especially cellulose, which facilitates faster cross-linking during the vulcanization process, resulting in a more efficient stress transfer and improved curing behaviour, making cellulose a more effective filler than rice straw in reducing pressing times and optimizing industrial production [41].

**Figure 4:**
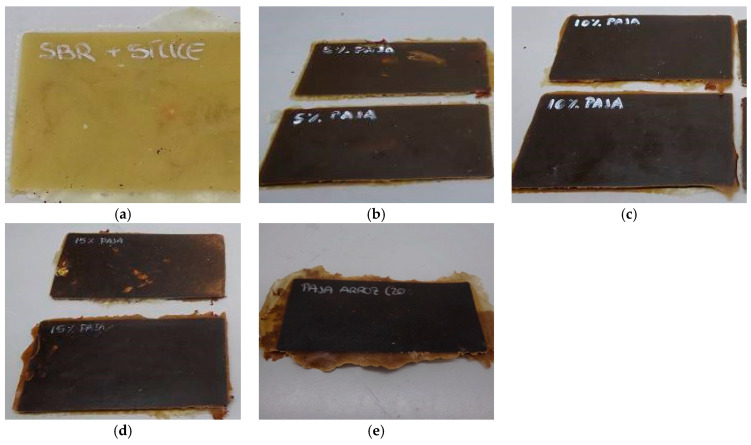
SBR with rice straw filler at (**a**) 0%, (**b**) 5%, (**c**) 10%, (**d**) 15%, and (**e**) 20%.

**Figure 5:**
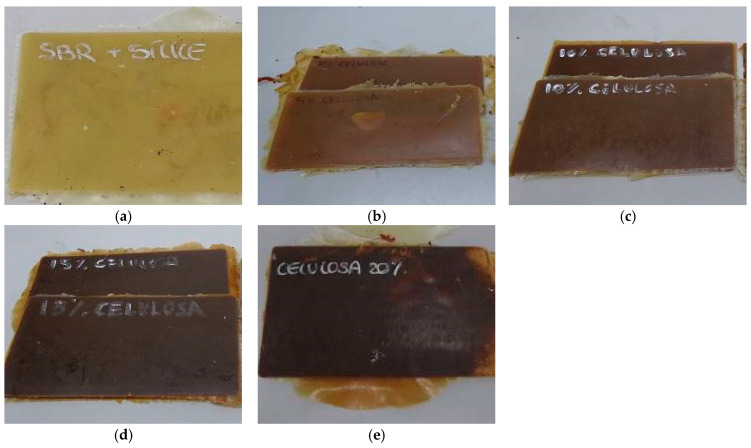
SBR with cellulose filler at (**a**) 0%, (**b**) 5%, (**c**) 10%, (**d**) 15%, and (**e**) 20%.

Thus, the obtained sheets are shown in the images of Figure 4 and Figure 5 for the two fillers tested.

The SBR sheets show notable differences in colour with the addition of rice straw and cellulose. SBR starts with a yellowish tone due to its base components. The addition of rice straw produces a darker compound compared to the incorporation of cellulose. This is due to the high temperatures during vulcanization, which can trigger the Maillard reaction between carbohydrates and amino acids, resulting in a darker material due to the formation of melanoidins [44,45,46].

In comparison, the sheets with cellulose also show darkening, but this effect is less pronounced until concentrations of 10–15%. Pure cellulose, derived from rice straw but without lignified components, has a lighter colour than that of whole rice straw. However, at higher concentrations (15% and 20%), cellulose also causes significant darkening. This effect is due to the possible thermal degradation of carbohydrates during vulcanization, which can trigger Maillard reactions, producing dark compounds that affect the final colouration of the compound [44,45,47].

### 3.2. Preparation of Bio-Based TPU with Rice Straw and Cellulose

**Figure 6:**
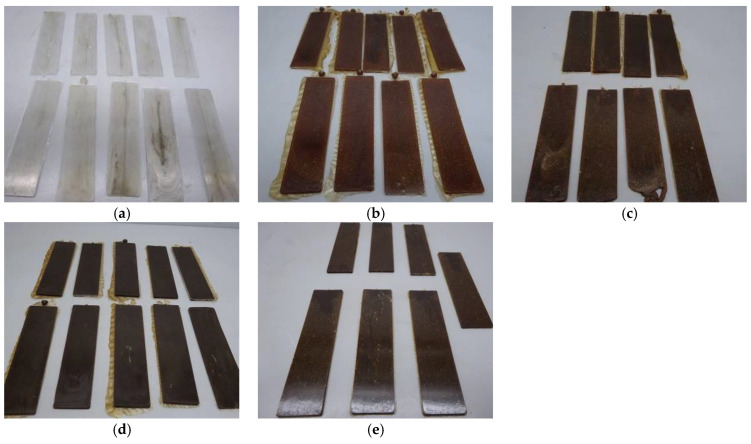
TPU with rice straw filler at (**a**) 0%, (**b**) 5%, (**c**) 10%, (**d**) 15%, and (**e**) 20%.

**Figure 7:**
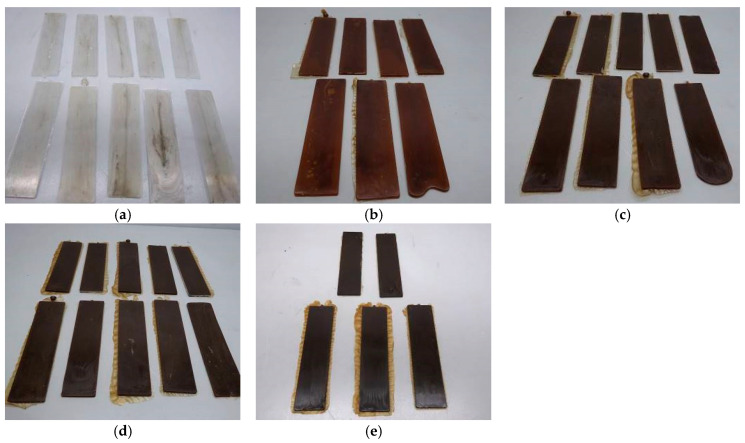
TPU with cellulose filler at (**a**) 0%, (**b**) 5%, (**c**) 10%, (**d**) 15%, and (**e**) 20%.

After preparing the mixtures and manually granulating them, they were mixed with the different amounts of filler to finally be moulded into plates through injection, which is shown in Figure 6 and Figure 7.

The TPU compounds also show similar colour changes to those of SBR, although they start with a lighter initial colour in comparison. On the one hand, as shown before, both filler samples progressively darken with increasing filler concentration. This darkening is due to the thermal degradation reactions of carbohydrates at high temperatures, producing dark compounds through caramelization and Maillard reactions, such as for SBR samples [44]. Because TPU is initially lighter than SBR, the colour change is more evident and rapid, showing colours ranging from light to dark brown at higher concentrations [46,47].

### 3.3. Characterization of Mechanical Properties

The mechanical properties of both SBR and TPU compounds filled with rice straw and cellulose were evaluated and compared. Various parameters, such as hardness, density, abrasion resistance, tensile strength, elongation, and tear resistance, were measured and analysed to assess the performance of these materials in footwear sole applications. Although no specific standard for casual footwear soles is available, the thresholds for mechanical properties in this study have been established through extensive industry experience and were based on UNE 59930 [48], which is only applicable to more stringent athletic footwear.

#### 3.3.1. SBR Compounds with Rice Straw

The mechanical properties of SBR compounds with varying percentages of rice straw are summarized in Table 6.

The analysis of the results obtained for SBR compounds with rice straw reveals several significant trends. First, the hardness of the compounds consistently increases with the addition of rice straw, from 56.8 Shore A in the non-filler compound to 67.4 Shore A in the compound with 20% rice straw. This increase is attributed to the rigid nature of rice straw, which acts as a reinforcement filler within the SBR matrix, increasing its stiffness and strength [49]. Density, on the other hand, shows slight fluctuations, indicating that the incorporation of rice straw does not significantly impact this property. This suggests uniform dispersion of the filler within the matrix, maintaining the structure of the compound without drastic changes in its overall density.

Abrasion resistance increases with the addition of rice straw, reaching a maximum allowable value at approximately 10%. Higher increments in rice straw content (15% and 20%) result in a deterioration in abrasion resistance, suggesting the existence of a threshold at approximately 10%. This behaviour might be explained by the increase in the stiffness of the material as the filler is incorporated [50]. It is worth noting that the values obtained for abrasion resistance in compounds with 15% and 20% rice straw exceed the recommended limit of 250 mm^3^, making them unsuitable for footwear sole applications according to industry standards.

In terms of tensile strength, an increase and improvement are observed at up to 10% rice straw, with a peak at 10.4 MPa, followed by a decrease to 7.9 MPa at higher concentrations. This suggests that rice straw reinforces the SBR matrix effectively at lower concentrations, likely due to improved interfacial adhesion between the filler and matrix up to this point. However, beyond 10%, the tensile strength decreases, possibly due to filler agglomeration and insufficient dispersion, which leads to weak spots in the material [51]. This trend is commonly observed with bio-based fillers such as rice straw and cellulose, as the hydrophilic nature of the filler can lead to poor adhesion to the hydrophobic rubber matrix, particularly at higher filler contents [52,53,54,55]. Additionally, compounds with 20% rice straw do not meet the minimum requirement of 8 MPa for footwear soles [33].

On the other hand, elongation at break exhibits a somewhat different trend. It increases initially, reaching a peak of 555.63% at 10% rice straw, suggesting improved flexibility up to this filler content. However, it decreases significantly at 15%, dropping to 444.4%, indicating that higher concentrations of rice straw reduce the material’s ability to stretch before breaking. This behaviour can be explained by the increased stiffness introduced by higher filler content, which restricts the movement of polymer chains and leads to premature rupture [56]. Despite this, all compounds meet the minimum elongation requirement of 400% for footwear soles.

Finally, tear resistance significantly improves with increasing rice straw content, reaching 21.3 N/mm at 20%, far exceeding the minimum requirement of 8 N/mm [57]. This increase suggests that rice straw enhances the material’s resistance to crack propagation, likely by reinforcing the matrix and distributing the stress more evenly across the material. Similar results have been observed in other studies where natural fillers enhance tear resistance, even if other properties such as tensile strength or abrasion resistance may be compromised at higher filler contents [56,58]. The observed increase in hardness and tear resistance with rice straw content may be related to the reduction in vulcanization times discussed previously. The faster formation of cross-links in the presence of rice straw likely contributes to the creation of a more rigid and tear-resistant matrix, which explains the enhanced mechanical properties in these aspects [41].

#### 3.3.2. SBR Compounds with Cellulose

The mechanical properties of SBR compounds with varying percentages of cellulose are summarized in Table 7.

The addition of cellulose to SBR compounds also shows a pattern of increasing hardness, although this increase is less pronounced than with rice straw. Hardness reaches its maximum in the compound with 15% cellulose, suggesting an improvement in the rigidity of the polymer matrix due to the incorporation of the filler. This increase in hardness may be linked to the accelerated vulcanization process observed with cellulose, as it acts as a nucleation site, promoting faster cross-linking and resulting in a stiffer matrix, as seen in the reduced pressing times from 4.0 min to 2.5 min with 20% cellulose content [41,59]. On the other hand, the density of SBR compounds with cellulose gradually increases, although not significantly, with the filler content, suggesting greater compaction of the polymer matrix. This increase in density could be beneficial for applications requiring denser and more compact materials.

The abrasion resistance of the compounds shows a similar trend to that observed with rice straw, with an initial increase reaching its maximum allowable point at approximately 10–15%, exceeding the threshold for higher percentages. Similar to rice straw, the rigid and interconnected structure formed during vulcanization does not necessarily improve abrasion resistance at higher filler contents, as the stiffness may lead to weaker wear performance [60]. This pattern indicates that cellulose incorporates well into the matrix up to a point, after which properties begin to deteriorate, likely due to the agglomeration of cellulose particles that create weak points.

Similar to rice straw, the tensile strength of SBR compounds with cellulose also improves with the addition of the filler up to 10%, indicating effective reinforcement of the matrix. However, at higher concentrations, strength decreases, suggesting that optimal dispersion is crucial to maximize this property. Elongation at break, in contrast, shows a general trend of decreasing with increasing cellulose content, except for the compound with 15%, where an increase is observed. This indicates that cellulose introduces stiffness and reduces the elasticity of the compound, although, at certain concentrations, it can improve the overall strength of the material. All compounds meet the minimum requirement of 400% elongation.

Tear resistance improves with increasing cellulose content, reaching a maximum at 15%. As with hardness, this improvement in tear resistance is also likely connected to the faster cross-linking in the presence of cellulose, which enhances the matrix’s ability to resist crack propagation [41,58]. This points to the idea that cellulose, like rice straw, reinforces the polymer matrix, although in a less marked way, increasing its ability to resist crack propagation, well above the minimum required value of 8 N/mm.

In summary, in SBR compounds, both rice straw and cellulose show improvements in hardness and density, although these improvements are more noticeable in rice straw. Moreover, abrasion resistance is better in cellulose, with lower values indicating greater durability. Additionally, tensile strength is slightly higher in rice straw compounds at 10%, although overall values are similar. Regarding elongation, it is higher in rice straw, indicating better elasticity, while tear resistance is significantly better in rice straw. This suggests that 10% rice straw could be the highest acceptable substitution, providing an optimal combination of mechanical properties.

#### 3.3.3. TPU Compounds with Rice Straw

The mechanical properties of TPU compounds with varying percentages of rice straw are summarized in Table 8.

The addition of rice straw to TPU compounds resulted in a steady increase in hardness, which reached a maximum of 90.6 Shore A in the compound with 20% rice straw. This increase can be attributed to the rigid nature of rice straw, which acts as a reinforcing agent that limits the deformation of TPU under applied force. The rice straw particles contribute to the overall stiffness of the matrix by restricting the mobility of the polymer chains and increasing the resistance to indentation [61,62]. The density of TPU compounds with rice straw remains relatively constant, with a notable increase only at the highest concentration (20%), suggesting that the filler dispersion is uniform at low and medium concentrations but may cause greater compaction at higher levels.

Abrasion resistance shows an initial increasing trend for up to 15% rice straw but decreases significantly at higher concentrations. This suggests that while rice straw initially enhances the compound’s wear resistance, the inherent nature of the filler as a plant-based material makes it less resistant to friction compared to pure TPU. The weaker bonding between the rice straw and the TPU matrix, compared to just the TPU itself, may lead to easier detachment of the filler particles during wear, contributing to higher material loss at higher concentrations [62]. This behaviour suggests that approximately 15% minimizes material durability without compromising other mechanical properties.

In terms of tensile strength, a decrease is observed as the rice straw content increases, indicating that the filler introduces weak points in the TPU matrix at higher concentrations. However, given that TPU is rich in hydroxyl groups, it is possible that the rice straw interacts with the polymer matrix, creating some level of bonding between the hydroxyl groups in both materials. These interactions could improve the filler dispersion and load transfer at lower concentrations, but at higher filler contents, issues like filler agglomeration or weak interfacial bonding may prevail, leading to the observed reduction in tensile strength [62,63]. This phenomenon is also reflected in elongation, where a progressive decrease is observed with increasing rice straw content. The reduction in compound elasticity could be related to the intrinsic rigidity of the filler. Despite the decrease, all compounds meet the minimum requirement of 7 MPa tensile strength and 400% elongation.

Regarding tear resistance, although there is some fluctuation, the values remain close to the baseline of the unfilled TPU. The small variations observed suggest that the inherent tear resistance of the TPU, which is already quite high, is not significantly affected by the incorporation of rice straw. This indicates that even at 20% filler content, the filler is not substantially disrupting the polymer’s ability to resist tear propagation. The rice straw may not be contributing significantly to improving tear resistance, but the TPU’s innate properties are robust enough to maintain this performance [64].

#### 3.3.4. TPU Compounds with Cellulose

The mechanical properties of TPU compounds with varying percentages of cellulose are summarized in Table 9.

The incorporation of cellulose in TPU compounds resulted in an increase in hardness, similar to the results obtained with rice straw. Hardness increased from 82.6 Shore A in the non-filler compound to 88.0 Shore A in the compound with 20% cellulose. This increase can be attributed to the rigid nature of cellulose, which, like rice straw, helps to reinforce the TPU matrix by restricting the movement of polymer chains and increasing the material’s resistance to deformation. On the other hand, the density of TPU compounds with cellulose shows a gradual increase with an increase in filler content, suggesting greater compaction and better dispersion of cellulose within the polymer matrix. The similar density of cellulose to TPU helps maintain a uniform structure at lower concentrations, but at higher percentages, the increased filler content likely leads to denser packing and less flexibility within the matrix.

In terms of abrasion resistance, TPU compounds with cellulose show a trend of gradually increasing until higher concentrations, with 15% being the maximum accepted point. The fibrous nature of cellulose may contribute to a higher rate of material loss under friction, as the fibres can detach more easily from the TPU matrix, leading to greater wear. Furthermore, poor interfacial bonding between cellulose and TPU could exacerbate this effect, making it easier for particles to detach during abrasion [62].

Tensile strength consistently decreases with increasing cellulose content, indicating that cellulose may introduce discontinuities in the TPU matrix, compromising its structural integrity, a critical parameter for its application as a footwear sole. However, at higher concentrations, the agglomeration of cellulose particles and weaker interfacial bonding may prevail, leading to a reduction in tensile strength [62,63]. This phenomenon is also reflected in elongation at break, where a progressive decrease is observed with increasing cellulose content, indicating a reduction in the compound’s elasticity. Despite the decrease, all cellulose compounds meet the minimum requirement of 7 MPa tensile strength and 400% elongation, although, from 15% onwards, properties decrease notably.

Finally, tear resistance decreases significantly with the addition of cellulose, especially at higher concentrations, where the increased rigidity of cellulose and potential agglomeration could introduce points of weakness in the matrix. These weak points reduce the material’s ability to resist crack propagation, leading to a significant drop in tear resistance at 20%. However, at lower concentrations, the inherent properties of TPU may mitigate these effects, maintaining tear resistance above the required thresholds [64]. Despite the inherent properties of TPU, the values are quite high regardless of cellulose concentration, exceeding the minimum required value; however, from 15%, the properties are diminished, as was the case with elongation, tensile strength, and abrasion.

In summary, when comparing both fillers for TPU compounds, hardness and density increase similarly, with no remarkable differences. Thanks to the inherent properties of TPU, abrasion resistance allows for any percentage of rice straw, while cellulose is acceptable up to 15% due to its tendency to increase wear at higher concentrations. Tensile strength is slightly better with rice straw, although both are comparable at lower filler percentages. The same occurs for elongation, with both showing better performance at lower percentages. Finally, tear resistance is consistently superior with rice straw at all percentages, likely due to its better integration into the TPU matrix and fewer points of weakness. Thus, due to the high inherent properties of TPU, and based on the results obtained, it is possible to add rice straw up to 20%, while incorporating cellulose is acceptable up to 15%, although lower percentages around 10% would be recommended for both as the properties do not diminish in the same way as for higher percentages.

The results obtained for both materials, fillers, and added percentages provide a solid basis for the use of rice straw and cellulose as fillers in SBR and TPU compounds. Identifying an optimal substitution threshold is crucial to maximize the desired mechanical properties. Therefore, an analysis of variance (ANOVA) based on a cross-factor experimental design was carried out, through which an optimal percentage of each filler for both materials could be determined. This analysis was performed by taking into account the mechanical properties of both materials and was developed after the thermal characterization results section of this article.

### 3.4. Thermal Analysis of TPU and SBR Compounds: Stability and Degradation Insight

In the production of shoe soles, thermal treatment of materials is a key part of the manufacturing process, as it influences the curing, durability, and performance of the final product. Studying the thermal properties of composites is therefore essential to ensure their stability and suitability for these applications. Typically, the temperatures involved in processing shoe soles range between 150 °C and 200 °C, depending on the specific material and technology used [65,66,67]. In terms of thermal stability, the TGA provides further insights into how the incorporation of both cellulose and rice straw influences the thermal degradation behaviour of TPU and compounds. Table 10 shows the derivative thermogravimetry (DTG) data for TPU with cellulose and rice straw fillers.

For TPU, as presented in Table 10, its degradation process is characterized by four distinct stages, the first with a peak corresponding to moisture loss and minor volatiles. In this discussion, particular attention is given to the third and fourth stages, which reflect the decomposition of the soft and hard segments of the polymer, respectively. However, in the cases where cellulose and rice straw are incorporated, there is a certain contribution from cellulose and lignin (or lignin residues in the cellulose filler sample) to these peaks, respectively, as they fall within the same temperature ranges. The inclusion of cellulose and rice straw modifies these degradation patterns due to the chemical interactions between the polymer matrix and fillers [68].

**Figure 8:**
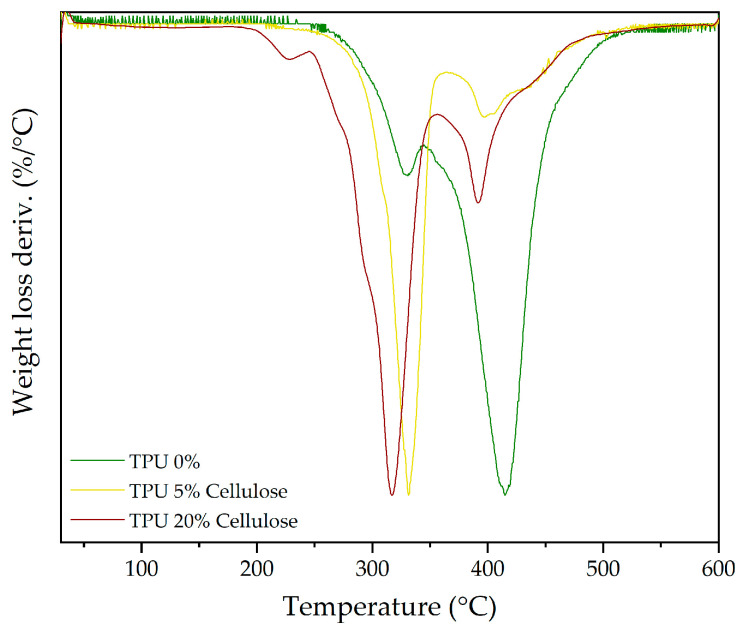
DTG curves for TPU composites with 0%, 5%, and 20% cellulose.

When examining the TPU reference sample (0%) compared to those incorporating different percentages of cellulose, significant shifts in the degradation behaviour of the polymer’s soft and hard segments are observed. Figure 8 illustrates that as the cellulose content increases from 5% to 20%, the soft segment peak (peak 3) shifts to higher weight loss values. For example, at 20% cellulose, the weight loss reaches 69.65%, compared to 21.99% in pure TPU. This increase suggests that the cellulose may disrupt the structure of the hard TPU segments, possibly softening these regions through interaction with the cellulose fibres. This could explain the shift in degradation behaviour, where the soft segments appear to gain prominence as the hard segments are weakened. Additionally, the enhanced thermal stability observed may result from the cellulose forming a more cohesive network with the polymer, redistributing the mechanical properties [69]. Additionally, the temperature at which these soft segments degrade tends to decrease with increasing cellulose content, indicating that higher cellulose concentrations may slightly disrupt the matrix and lead to earlier degradation at 20%.

A similar trend is observed regarding the hard segments (peak 4), though the effect is more pronounced at higher cellulose concentrations. The weight loss decreases from 77.71% in pure TPU to 29.95% at 20% cellulose, suggesting that cellulose contributes to forming a char residue that stabilizes the degradation process. However, there is also a shift to lower temperatures for the hard segments as the cellulose content increases, indicating that the presence of cellulose alters the interaction between the polymer chains in this region, possibly reducing the thermal stability of these segments as the cellulose becomes more integrated within the matrix.

**Figure 9:**
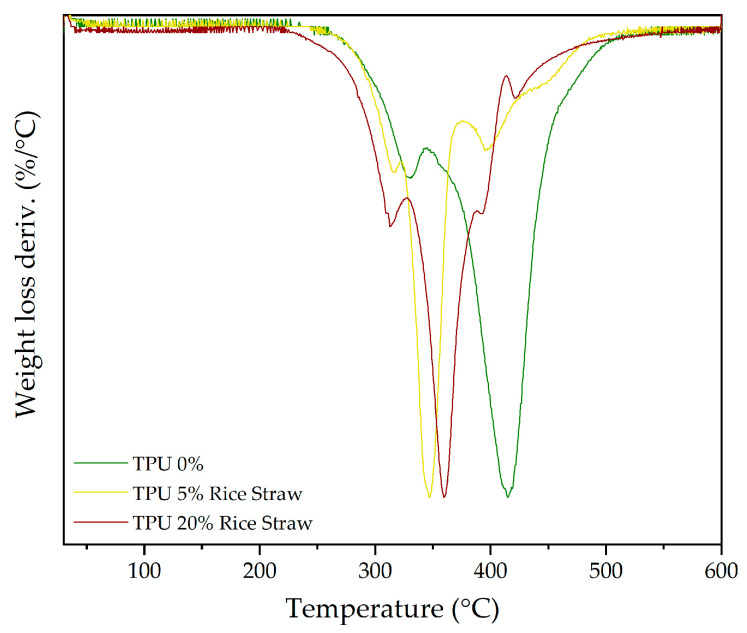
DTG curves for TPU composites with 0%, 5%, and 20% rice straw.

In the case of rice straw, the degradation behaviour follows a slightly different trend, as shown in Figure 9. The third degradation peak, which corresponds to the soft segments, shifts to higher weight losses, which is similar to the cellulose composites but with an additional contribution from hemicellulose. Furthermore, this peak shifts to higher temperatures with increasing rice straw content, with a more significant difference of about 30 degrees at 20% rice straw compared to the pure TPU sample. This behaviour suggests that rice straw, now with its hemicellulose component, interacts with the soft segments, stabilizing them and delaying their degradation. In that sense, the addition of rice straw introduces a new degradation stage (peak 2) attributed to the hemicellulose present in the rice straw, which is absent in the cellulose samples due to the purification process. Both cellulose and hemicellulose, which are present in rice straw, contain hydroxyl (OH) groups that interact with the soft and hard segments of TPU. Specifically, the OH groups can interact with the isocyanate groups in the hard segments of TPU, facilitating a conversion of some hard segments into soft segments. This conversion leads to a higher proportion of soft segments, altering the thermal degradation behaviour by stabilizing them and delaying their degradation [70,71].

For the hard segments (peak 4), the use of rice straw leads to a reduction in the weight loss values, similar to the effect observed with cellulose. However, the degradation of the hard segments is slightly displaced, especially at 5% rice straw, where lignin likely plays a role in this temperature range. Lignin, another major component of rice straw, decomposes over a wide temperature range, and its interaction with the hard segments of TPU appears to induce minor destabilization. The effect is more notable in the 5% composite, where lignin could be interfering with the cross-linking of the hard segments, reducing the char-forming efficiency. The reduction in weight loss at 20% rice straw is more moderate compared to cellulose, indicating that rice straw, while improving thermal stability, may not be as effective as cellulose in forming a stabilizing char residue for the hard segments.

These results indicate that while both cellulose and rice straw enhance the thermal stability of TPU composites, they do so through different mechanisms. Cellulose primarily reinforces the soft segments, delaying their degradation by forming strong hydrogen bonds with the polymer chains and promoting cohesive interaction within the matrix, while also contributing to a more stable degradation process for the hard segments. Rice straw, on the other hand, introduces additional degradation stages related to hemicellulose and lignin content, which not only stabilizes the soft segments through chemical interactions but also introduces new degradation pathways that alter the behaviour of both soft and hard segments. Both fillers interact differently with the polymer matrix, influencing the thermal behaviour in a more complex manner [72].

Following the thermal stability results discussed for TPU, the TGA of the SBR composites with cellulose and rice straw presents a more homogeneous degradation pattern. SBR, being a copolymer of styrene and butadiene, lacks the clear distinction between soft and hard segments seen in TPU. Instead, SBR behaves as a more uniform elastomer, which helps explain the minimal impact observed on its thermal behaviour upon adding fillers. The DTG results, presented in Table 11, show the decomposition stages and their respective weight loss percentages.

**Figure 10:**
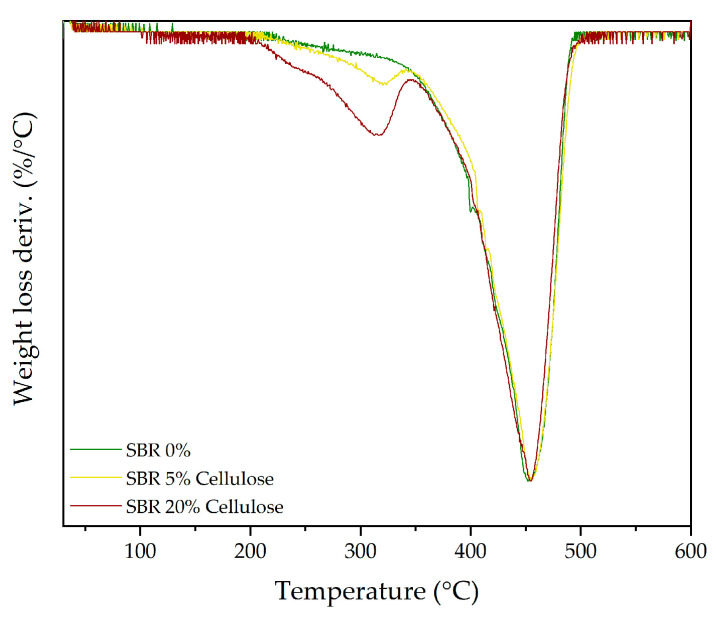
DTG curves for SBR composites with 0%, 5%, and 20% cellulose.

Figure 10 presents the DTG curves for SBR composites with 0%, 5%, and 20% cellulose. The primary degradation peak (peak 4), corresponding to the breakdown of the SBR backbone, remains largely unchanged regardless of the amount of cellulose incorporated. This consistency suggests that the addition of cellulose does not significantly disrupt the polymer structure of SBR. Instead, cellulose acts primarily as a filler, with minimal interaction with the SBR matrix [73]. However, the contribution of cellulose to the overall degradation process becomes more apparent as the filler content increases. As shown in Figure 10, at 20% cellulose, a noticeable increase in weight loss is observed in the third peak, which corresponds to the degradation of the cellulose itself. In pure SBR, this peak is almost negligible, but it grows substantially with higher cellulose content, reaching its maximum at 20%. Notably, while this peak becomes more prominent, it also shifts slightly to the left, indicating a minimal decrease in the degradation temperature—approximately 5 °C—which may indicate minor interactions between the hydroxyl groups of cellulose and the SBR matrix. Although these interactions are not strong enough to substantially alter the thermal stability of the SBR backbone, they may facilitate the earlier degradation of the composite by weakening localized regions of the polymer–filler interface.

**Figure 11:**
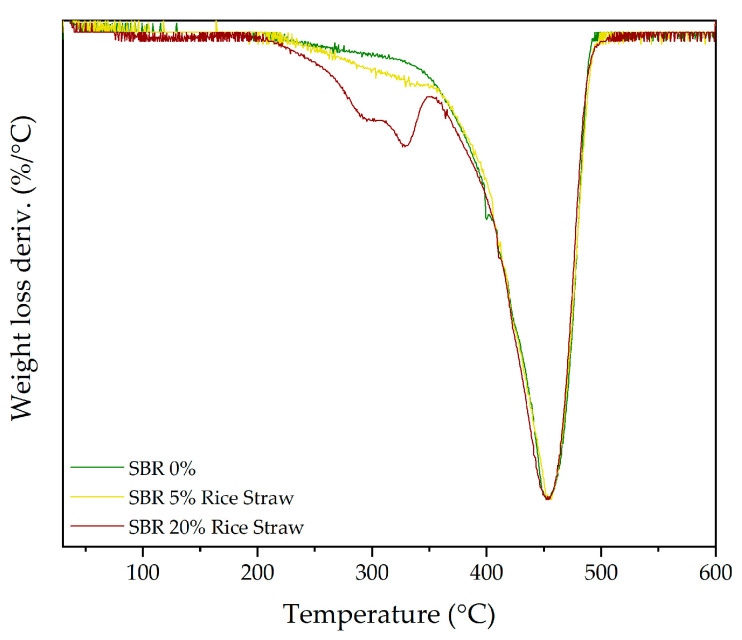
DTG curves for SBR composites with 0%, 5%, and 20% rice straw.

On the other hand, Figure 11 presents the DTG curves for SBR composites with 0%, 5%, and 20% rice straw. A similar trend is observed when compared to SBR composites using cellulose filler, although the presence of hemicellulose introduces additional complexity. The primary degradation peak of SBR (peak 4) remains dominant, not changing in weight loss or position, indicating once again that the SBR backbone is not significantly affected by the addition of fillers. However, the introduction of rice straw, particularly at 20%, brings forth new degradation stages. The second peak (peak 2), which corresponds to the degradation of hemicellulose, becomes increasingly visible as the rice straw content increases. This peak is subtle at 5% rice straw, but by 20%, it is clearly distinguishable, highlighting the significant contribution of both hemicellulose and cellulose to the degradation process.

In contrast, the peak corresponding to lignin within the rice straw (peak 4) remains largely undetectable in the TGA curves. This is likely due to the overlapping degradation of the SBR matrix, which occurs in the same temperature range as the decomposition of these components. The large contribution of the SBR backbone to the overall degradation profile effectively masks the smaller contributions from lignin, making it indistinguishable in the thermograms.

These findings suggest that both cellulose and rice straw act primarily as reinforcing fillers within the SBR matrix, with their main contributions being the introduction of additional degradation stages rather than altering the core thermal behaviour of SBR itself. The SBR backbone remains stable, with only minor shifts or reductions in thermal stability, as seen in the slight leftward shift of the cellulose degradation peak. Overall, the role of these fillers appears to be one of passive reinforcement rather than active modification of the degradation process.

After assessing the thermal degradation behaviour of both TPU and SBR through TGA, the next step involves exploring their thermal transitions using DSC. This analysis will focus on the crystallization and melting behaviour of the materials, providing insights into how the addition of cellulose and rice straw influences their thermal properties. First, the DSC behaviour of TPU will be discussed, followed by an analysis of SBR.

**Figure 12:**
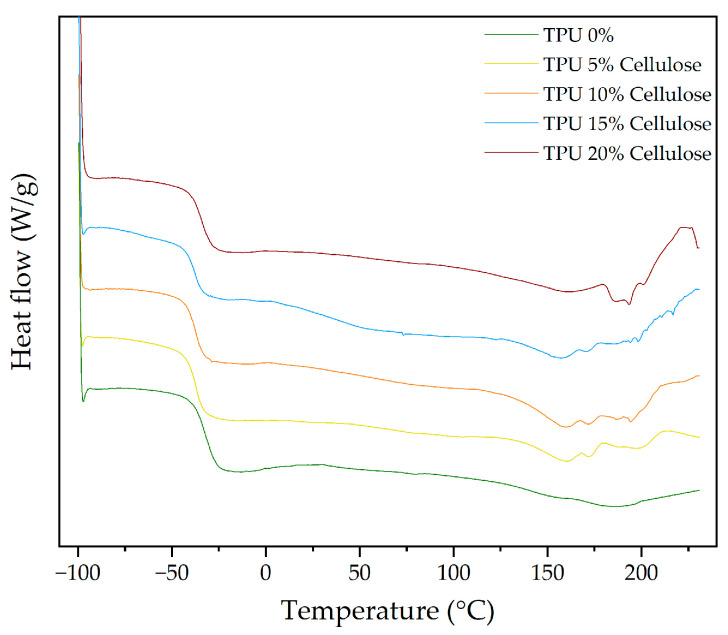
DSC curves for TPU composites with different cellulose contents.

As summarized in Table 12, the DSC thermograms reveal several key thermal transitions, including the glass transition temperature (T_g_) and melting temperatures (T_m_), for TPU composites with different cellulose contents. Figure 12 presents the DSC curves for TPU composites with varying cellulose concentrations. In the case of pure TPU, a well-defined melting peak is observed at 159.78 °C, alongside a glass transition temperature (T_g_) of −38.03 °C. As cellulose content increases, the melting behaviour changes, with T_m_ decreasing slightly and reaching 155.76 °C at 15% cellulose. This shift suggests that cellulose disrupts the crystalline regions of TPU, resulting in a reduction in crystallinity and promoting more amorphous behaviour [74].

Moreover, as seen in Figure 12, the T_g_ remains relatively stable at lower cellulose concentrations but starts to shift towards higher temperatures at 20% cellulose. This indicates a stronger interaction between cellulose and the amorphous regions of the TPU matrix, leading to increased rigidity and a delayed glass transition. This may be attributed to the hydrogen bonding between the hydroxyl groups in cellulose and the urethane groups in TPU, which could limit the flexibility of the soft segments. This behaviour is consistent with the results observed in TGA, where higher cellulose content stabilized the soft segments of the polymer matrix [70,71].

Figure 12 also shows the emergence of additional melting peaks at higher temperatures (T_m2_ and T_m3_) in samples with 5%, 10%, and 15% cellulose, which disappear at 20%. These new peaks suggest that cellulose induces the formation of additional crystalline domains that melt at higher temperatures. The presence of these new peaks, alongside the shift in T_m1_, points to structural heterogeneity introduced by cellulose within the TPU matrix.

ΔH_mT_, which reflects the energy associated with the melting process indicating the energy required to melt the crystalline regions of the material, decreases significantly as cellulose content increases, as shown in Table 12. For pure TPU, the enthalpy is −7.45 J/g, while for 20% cellulose, it drops to −10.15 J/g. This reduction in enthalpy further confirms the decrease in overall crystallinity as cellulose is incorporated, indicating that the polymer matrix becomes more amorphous with increasing cellulose content.

**Figure 13:**
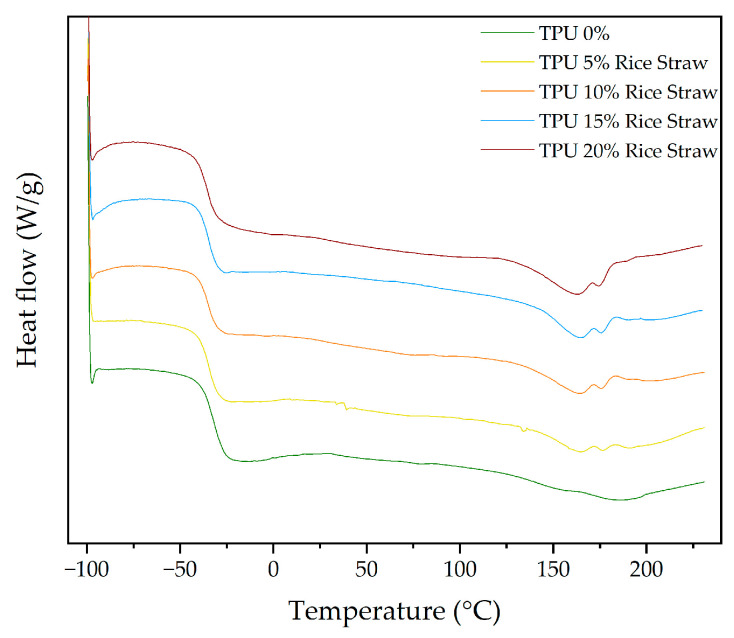
DSC curves for TPU composites with different rice straw contents.

Figure 13 displays the DSC curves for TPU composites with different rice straw contents, and, in Table 12, the DSC thermograms reveal several key thermal transitions, including the glass transition temperature (T_g_) and melting temperatures (T_m_), for TPU composites with different rice straw contents. The glass transition temperature (T_g_) of pure TPU (0% RS) is recorded at −38.03 °C, indicating the flexibility and mobility of the soft segments. With the incorporation of rice straw, there is a slight increase in Tg, ranging from −36.13 °C to −35.01 °C. This change suggests that the rice straw introduces some restrictions to the mobility of the soft segments. Similar to other natural fibres like cellulose, this behaviour can be attributed to hydrogen bonding between the hydroxyl groups of rice straw and the urethane groups in TPU, leading to a slight stiffening of the amorphous regions of the TPU matrix. The melting temperature (T_m_) shows an initial increase at 5% rice straw, followed by a gradual decrease at higher concentrations, reaching 161.09 °C at 20%.

Two additional melting peaks (T_m2_ and T_m3_) appear at higher temperatures, similar to the behaviour observed with cellulose. These peaks suggest that rice straw, like cellulose, introduces structural heterogeneity in the TPU matrix. The total enthalpy of fusion decreases with rice straw addition, though less dramatically than with cellulose, suggesting that rice straw reduces crystallinity, but to a lesser extent.

These DSC findings align with the TGA results, showing that both cellulose and rice straw modify the thermal transitions of TPU, reducing overall crystallinity and introducing new melting domains.

**Figure 14:**
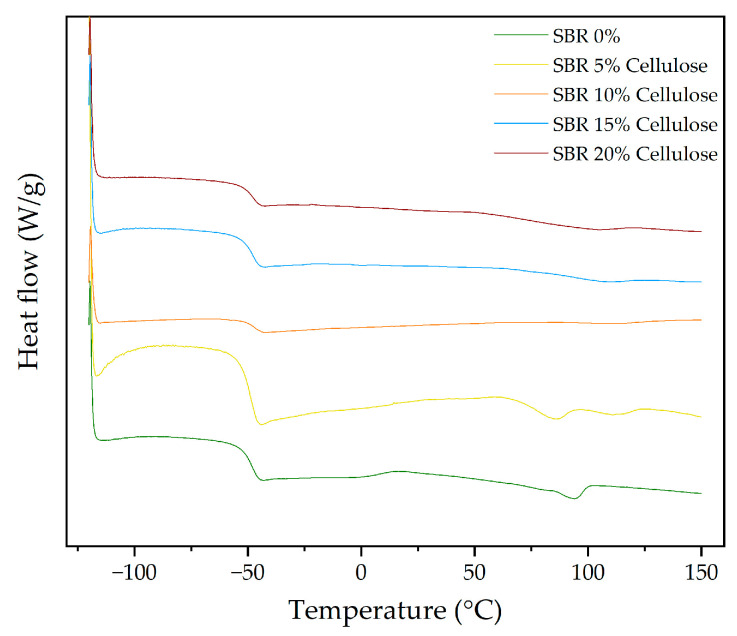
DSC curves for SBR composites with different cellulose contents.

On the other hand, the DSC results for SBR composites with varying amounts of cellulose and rice straw are now discussed, focusing on how these fillers affect the thermal transitions of the material. As summarized in Table 13, the DSC thermograms reveal several key thermal transitions for SBR composites with different cellulose contents, including the glass transition temperature (T_g_), crystallization temperature (T_c_), and melting temperatures (T_m_). Figure 14 presents the DSC curves for SBR composites with different cellulose contents. The pure SBR sample shows a glass transition temperature (T_g_) of −48.85 °C and two melting peaks at 65.79 °C and 94.27 °C. The T_g_ remains relatively stable across all cellulose concentrations, with only minor shifts. The most significant shift is seen at 10% cellulose, where T_g_ rises slightly to −47.69 °C, suggesting a modest interaction between the cellulose and the amorphous regions of the SBR matrix, slightly restricting chain mobility. On the other hand, the pure SBR sample shows a crystallization temperature (T_c_) at 16.14 °C, which disappears in all samples containing cellulose. This absence of T_c_ in the filled samples suggests that the addition of cellulose disrupts the crystallization process, likely due to the increased heterogeneity in the polymer matrix. This could be due to the hydrogen bonding between the hydroxyl groups of cellulose and the double bonds in the polybutadiene segment of SBR. Cellulose interferes with the regular packing of the polymer chains, preventing the formation of well-defined crystalline regions during cooling, which is why T_c_ is not observed.

The melting behaviour changes more significantly with the addition of cellulose. As observed in Figure 14, the melting temperature (T_m1_) of SBR decreases consistently as the cellulose content increases, reaching 77.01 °C at 10% cellulose. The second melting peak (T_m2_) shifts to 113.84 °C at 10% cellulose, indicating that cellulose disrupts the crystallinity of the SBR matrix and introduces new crystalline regions that melt at higher temperatures.

ΔH_mT_ decreases slightly with increasing cellulose content. In the pure SBR sample, ΔH_mT_ is −2.13 J/g, while at 20% cellulose, it drops to −2.40 J/g. This modest reduction in enthalpy points to a slight decrease in crystallinity, likely due to the increasing density of cellulose within the matrix, leading to a more amorphous structure, which is consistent with the TGA results, where cellulose introduced new degradation peaks without significantly affecting the thermal stability of the SBR backbone.

**Figure 15:**
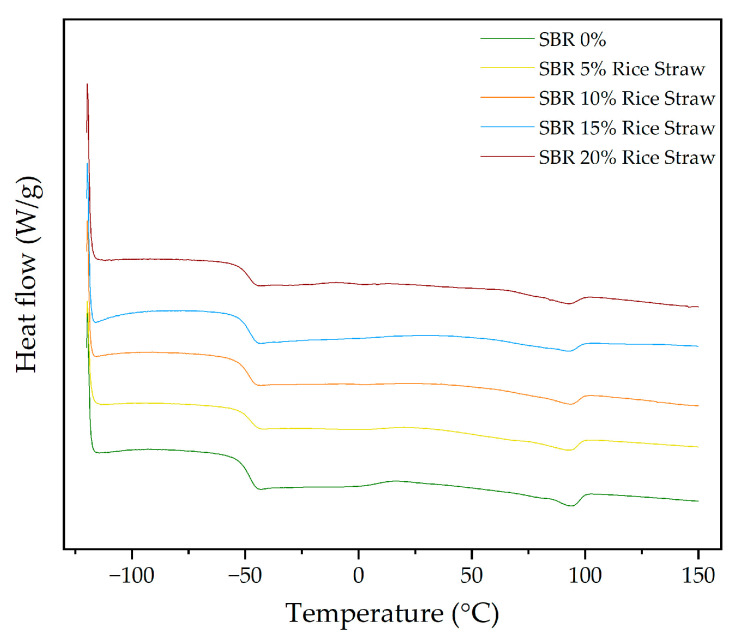
DSC curves for SBR composites with different rice straw contents.

Figure 15 shows the DSC curves for SBR composites with different rice straw contents. The glass transition temperature (T_g_) remains quite stable, with minor fluctuations. The T_g_ is −48.85 °C in pure SBR and decreases slightly to −49.33 °C at 15% rice straw, indicating minimal impact on the amorphous regions of the SBR matrix. In the case of crystallization temperature (T_c_), it appears at 23.94 °C in the 5% rice straw sample, suggesting that rice straw promotes crystallization during cooling at lower concentrations. However, T_c_ disappears in the 10%, 15%, and 20% samples, which is likely due to the increased structural complexity introduced by the rice straw that disrupts crystalline formation, similar to the effect seen with cellulose.

The melting behaviour of SBR with rice straw shows more notable changes. The melting temperature (T_m1_) increases significantly with the addition of rice straw, rising to 92.38 °C at 5% rice straw, as shown in Figure 15. This suggests that rice straw may act as a nucleating agent at lower concentrations, enhancing the crystallinity of the SBR matrix. However, at higher concentrations (20%), T_m1_ decreases slightly to 91.79 °C, indicating that rice straw begins to disrupt the crystalline regions of SBR at these higher levels.

ΔH_mT_ increases significantly from −2.13 J/g in pure SBR to −4.25 J/g at 5% rice straw, reflecting an initial increase in crystallinity. However, as the rice straw content increases, ΔH_mT_ gradually decreases to −1.87 J/g at 20%, indicating a reduction in crystallinity at higher concentrations, which aligns with the TGA findings.

ΔH_c_, representing the enthalpy of crystallization, is only observed in the pure SBR and the sample with 5% rice straw, indicating that crystallization occurs at these concentrations. However, at higher rice straw contents (10%, 15%, and 20%), the crystallization disappears. This suggests that the addition of rice straw at higher levels disrupts the polymer chain alignment, leading to an increase in amorphous regions and preventing detectable crystallization.

In summary, both cellulose and rice straw modify the thermal transitions of SBR, with cellulose slightly decreasing the melting temperatures and rice straw initially increasing them. The introduction of new crystalline domains and the reduction in crystallinity observed in the enthalpy changes are consistent with the TGA results, where both fillers introduced complexity into the degradation and thermal stability of SBR.

### 3.5. Statistical Analysis Results

To identify the significant effects of the factors and their interactions, an analysis of variance (ANOVA) was used. It was observed that both the type of material and the amount of filler were significant factors in several of the measured properties.

Initially, it is essential to note that the most suitable properties for application as a footwear component were observed in the samples with the initial formulation, i.e., those without any filler. This finding was expected, as materials in their original formulation usually exhibit the best mechanical properties. However, the objective of this study was not simply to identify the material with the most suitable properties in its natural state, but to explore how the incorporation of fillers can affect the properties of these materials, thus contributing to the existing literature and providing added value that can be critical for specific future applications, reducing economic and environmental impact by decreasing the consumption of virgin raw materials and substituting them with a substance initially considered as waste.

The *p*-value results obtained from the ANOVA for abrasion resistance, tensile strength, elongation, and tear resistance with respect to the material, filler, and added quantity are shown in Table 14, where A represents the material, B the filler, and C the filler quantity. In the case of tensile strength, an interaction was found between material and filler quantity, indicating that the optimal conditions for an SBR are very different from those for TPU. Therefore, the optimal filler formulation for both SBR and TPU can be selected. On the other hand, for the rest of the variables, there are no significant interactions in any case, so even though SBR and TPU are very different materials, the behaviour of the filler and the added quantity is similar for both. Therefore, these interactions have been ignored, thus increasing the residual factor and obtaining more reliable results for the analysis [75].

**Figure 16:**
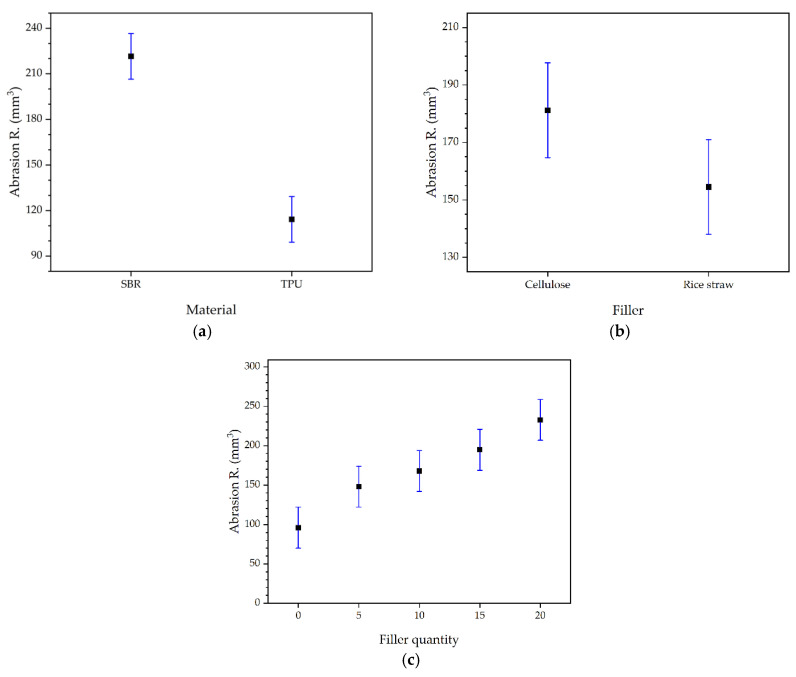
Mean values and 95% LSD intervals for abrasion resistance for the (**a**) material, (**b**) filler, and (**c**) filler quantity.

In the case of abrasion resistance, the ANOVA results showed that the type of material (*p* = 0.0000) and the quantity of filler (*p* = 0.0012) were significant factors, while the type of filler was not (*p* = 0.1102). This indicates that although the choice between TPU or SBR significantly impacts abrasion resistance, the nature of the filler (cellulose or rice straw) does not have a notable effect. However, the quantity of filler does significantly influence this property. Figure 16 shows the mean values and 95% LSD intervals for abrasion resistance, clarifying the influence of the filler quantity. The results show that increasing the filler quantity from 0% to 5% does not present significant statistical differences in abrasion resistance, which is positive as lower abrasion resistances imply greater durability. However, further increases to 10%, 15%, and 20% do not offer significant improvements and may even worsen abrasion resistance, although, in the case of 10% addition, it does not present significant differences with 5%, but it does with the initial formulation sample. This behaviour suggests that there may be an optimal threshold at approximately 5–10% of addition, where more sustainable formulations can be obtained without compromising material properties.

**Figure 17:**
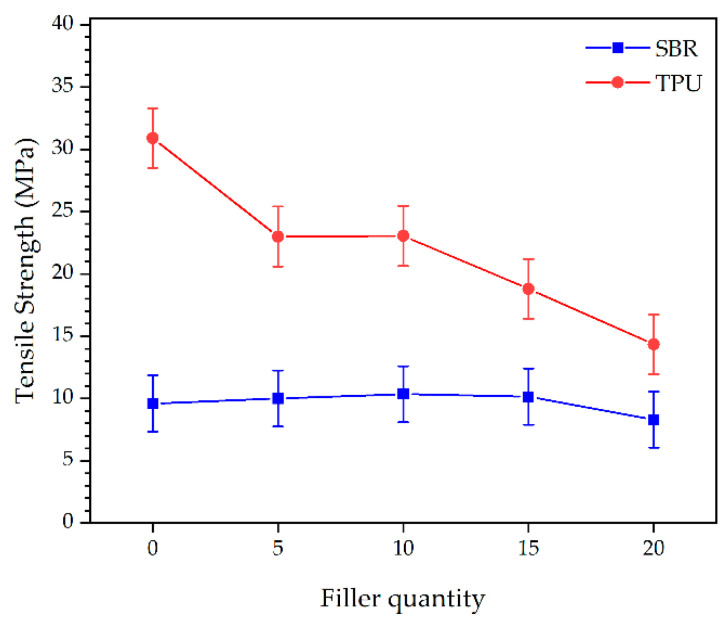
Interaction graph of the mean values and 95% LSD intervals for tensile strength results.

Regarding tensile strength, the results indicated that, as in the case of the abrasion resistance, both the type of material (*p* = 0.0000) and the quantity of filler (*p* = 0.0010) are significant factors. Again, the type of filler was not significant (*p* = 0.5125). In the case of tensile strength, there is an interaction between material and filler quantity, as shown in Table 14, so in order to observe the differences between samples, it is necessary to refer to the interaction graph of the mean values and 95% LSD intervals, shown in Figure 17. It is notably observed that TPU has significantly higher tensile strength compared to SBR, which stems from the intrinsic properties of the material itself. Additionally, in the case of TPU, the filler quantity also showed significant differences between 0% and the rest of the added quantities. It is important to emphasize that although the initial formulation offers the best properties, the addition of fillers can modify these properties usefully for specific applications. For instance, in the footwear sector, research focusing on natural rubber soles demonstrated that adjusting filler/plasticizer fractions can optimize properties like impact force absorption and hardness, with higher plasticizer levels reducing hardness and impact force while increasing energy dissipation, and increased filler content improving these properties [76,77,78]. Thus, a small reduction in properties concerning the optimum is permissible considering the cost reduction and added value contribution [79,80]. Therefore, after observing the statistical analysis results, the only TPU sample with notable differences from the rest is the 20% filler, with results closest to the initial formulation sample for 10% TPU and optimal results at all added percentages in SBR.

**Figure 18:**
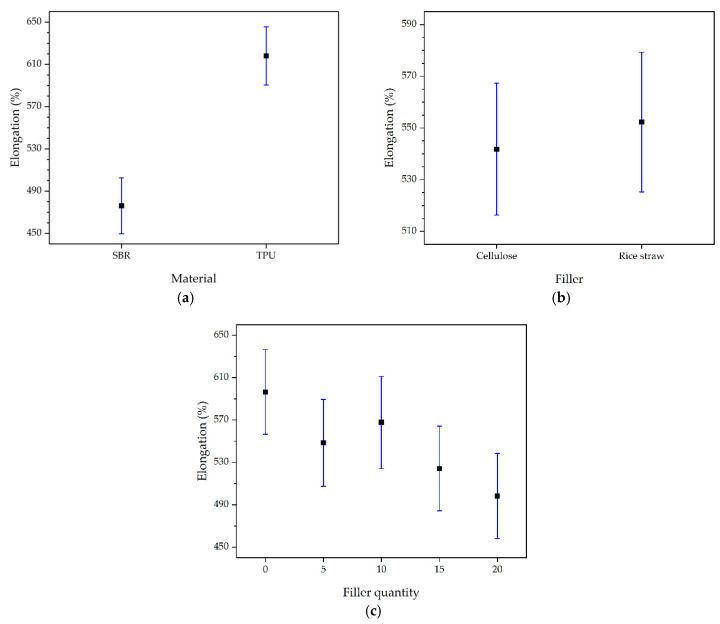
Mean values and 95% LSD intervals for elongation for the (**a**) material, (**b**) filler, and (**c**) filler quantity.

Figure 18 shows the mean values and 95% LSD intervals for elongation, where only the type of material has a significant effect (*p* = 0.0001), while the type of filler (*p* = 0.6684) has no significant effects. Regarding the filler quantity (*p* = 0.1519), different percentages do not present significant effects among themselves, although, when compared to the initial formulation material, the differences are not significant until a 20% substitution.

**Figure 19:**
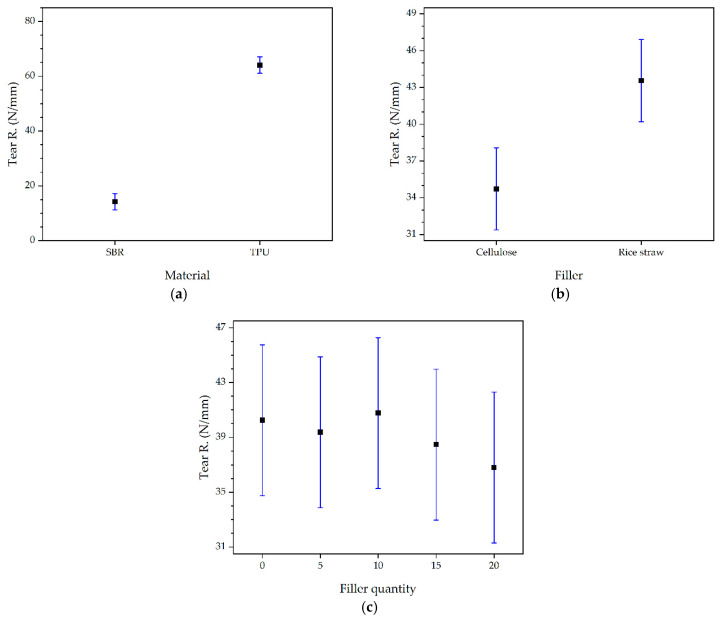
Mean values and 95% LSD intervals for tear resistance for the (**a**) material, (**b**) filler, and (**c**) filler quantity.

Regarding tear resistance, both the type of material (*p* = 0.0000) and the type of filler (*p* = 0.0166) were significant factors, while the filler quantity was not significant (*p* = 0.9386). The 95% LSD intervals for tear resistance are shown in Figure 19. Among the fillers, rice straw provided better tear resistance than cellulose, suggesting that this filler may be more effective in applications where tear resistance is critical, such as protective clothing, construction products, geotextiles, and athletic footwear soles, work boots, or safety footwear [81,82]. Although in the rest of the properties, the type of filler was not decisive, the tests using rice straw were slightly higher in all cases, which, together with the tear resistance results, indicates that the optimal formulations for this study, especially when good tear resistance properties are desired, are those using rice straw. The superiority of rice straw can be attributed to its lignified nature, providing additional rigidity and strength to the materials. This suggests that while cellulose extraction from rice straw is a laborious process and has been successfully optimized in previous research [21], the importance of pure cellulose should not be overlooked, as it can offer different and valuable properties in other applications, such as in the manufacture of sustainable membranes for supercapacitors, reinforced corn films, and chitosan biocomposites [83,84,85].

Thus, after approaching the statistical analysis for each property, it was confirmed that TPU inherently offers superior mechanical properties compared to SBR, which is consistent with findings in the literature. However, these differences arise from the inherent characteristics of each material [67,86,87]. The analysis has provided valuable insights into how the type and quantity of fillers influence key properties of each of them, particularly abrasion and tensile strength, offering practical guidance for optimizing formulations for future applications.

When it comes to the fillers used, the differences are not always significant, although rice straw tends to provide superior values compared to cellulose, making it the preferable option despite the relevance of extracting cellulose. On the other hand, the amount of filler did have a notable impact on abrasion and tensile strength. This suggests that while the nature of the filler is less critical, the amount of filler can be optimized to find a balance between obtained properties and added value to the product. Analysing all the results globally, it is established that the substitution of up to 10% rice straw allows for adding value to our product without significantly reducing some properties.

This study highlights the potential of incorporating lignocellulosic materials (such as rice straw) into TPU and SBR formulations, adding value while reducing the reliance on synthetic materials. This not only reinforces the sustainability of the solutions but also provides a foundation for future research aimed at developing more eco-friendly formulations by incorporating waste materials, thereby reducing dependence on fossil-based resources without compromising the final properties of the compounds [88,89,90,91].

The focus on rigorous statistical analysis through an ANOVA has allowed us to clearly identify the most influential factors and relevant interactions, providing a deep understanding of how to analyse the properties of these composite materials. The justification for including both TPU and SBR in the same analysis lies in the need for a global comparative vision, allowing for the determination of optimal conditions for each material and offering practical and applicable recommendations in the footwear industry and other sectors using these materials. This approach ensures that despite significant differences between materials, valuable and practical conclusions can be obtained to improve the properties of each material type through the optimization of the amount and type of filler used.

## 4. Conclusions

This study aimed to develop materials suitable for the production of shoe soles, investigating the effect of incorporating cellulose and rice straw as fillers into TPU and SBR compounds, with a focus on understanding how these natural fillers influence mechanical, thermal, and structural properties. Both fillers introduced significant changes in the performance of the polymer matrices, affecting key properties such as tensile strength, elongation, tear resistance, and thermal stability.

For TPU, the incorporation of cellulose and rice straw had distinct effects on mechanical properties. Cellulose increased the stiffness of the TPU, as reflected in higher hardness values (up to 88.0 Shore A at 20% cellulose). Tensile strength decreased from 30.9 MPa in pure TPU to 10.6 MPa at 20% filler content, suggesting filler agglomeration at higher levels. Rice straw also increased hardness, reaching 90.6 Shore A at 20% filler, but tensile strength showed a more gradual decline, from 30.9 MPa to 18.1 MPa, at 20% filler. The reduction in tensile strength for both fillers can be attributed to the filler particles disrupting the polymer matrix, leading to weak points. However, tear resistance remained strong, especially with rice straw, which improved tear resistance to 73.6 N/mm at 20% filler content, suggesting that this filler provides additional reinforcement in TPU matrices.

In the case of SBR, the mechanical properties showed similar trends with both cellulose and rice straw. Hardness increased with filler content, reaching 67.4 Shore A with 20% rice straw and 63.0 Shore A with 15% cellulose. In terms of tensile strength, rice straw performed better than cellulose, reaching 10.4 MPa at 10% rice straw before declining at higher filler levels, while cellulose maintained a more moderate tensile strength of 10.3 MPa at the same level. Abrasion resistance, however, showed that 10% filler content was optimal for both cellulose and rice straw, as it did not exceed the industry threshold of 250 mm^3^. At higher filler contents (15–20%), abrasion resistance worsened, with values exceeding 250 mm^3^, making these higher concentrations less suitable for footwear soles.

The thermal properties of the TPU and SBR composites were also significantly influenced by the fillers. In TPU, both cellulose and rice straw delayed the degradation of the soft segments, improving thermal stability. Cellulose enhanced the stability of the soft segments, increasing the weight loss to 69.7% at 20% cellulose, while rice straw contributed additional degradation stages due to its hemicellulose and lignin content. In SBR, both fillers introduced their corresponding new degradation stages but had less effect on the core thermal stability of the material itself compared to TPU.

The statistical analysis confirmed that the type of material and the amount of filler were significant factors affecting mechanical properties. ANOVA results showed that material type and filler quantity had significant impacts on properties such as abrasion resistance and tensile strength, while filler type was less critical. For both TPU and SBR, 10% filler content appeared to provide the best balance between maintaining material properties and introducing sustainable fillers, with rice straw proving particularly effective at enhancing tear resistance.

In conclusion, the materials obtained in this study, particularly TPU with 10% rice straw, show promising potential for shoe sole applications due to their balanced mechanical and thermal properties. Further development could involve optimizing these composites or exploring additional modifications to improve performance further, ensuring optimal properties for specific footwear requirements.

## Figures and Tables

**Table 1:** Formulation of thermoset SBR compounds with silica.

Compounds	Amount (%)
SBR 1502	69.11
Silica	20.73
Rice straw/cellulose	--
TDAE oil	3.46
Zinc oxide	1.38
Stearic acid	1.38
PEG	1.24
MBTS	0.83
TMTD	0.28
DPG	0.55
Sulphur	1.04

**Table 2:** Formulation of thermoplastic TPU compounds with rice straw or cellulose filler.

Compounds	0%	5%	10%	15%	20%
TPU-wantane	60.9	57.9	54.8	51.8	48.7
Rice straw/Cellulose	--	3.1	6.1	9.1	12.2

**Table 3:** Rheometric study results of SBR compounds with rice straw filler.

Rice Straw (%)	S’Maximum (Ib-in)	S’Minimum (Ib-in)	tc 90 (min)	Scorch Time (ts 0.5) (min)	ts 2 (min)
0	13.06	2.21	2.78	1.51	1.76
5	13.55	1.97	2.12	1.06	1.24
10	13.26	2.33	1.80	0.94	1.10
15	14.95	2.49	1.36	0.74	0.85
20	14.51	2.97	1.49	0.76	0.89

**Table 4:** Rheometric study results of SBR compounds with cellulose filler.

Cellulose (%)	S’Maximum (Ib-in)	S’Minimum (Ib-in)	tc 90 (min)	Scorch Time (ts 0.5) (min)	ts 2 (min)
0	13.06	2.21	2.78	1.51	1.76
5	14.57	2.16	1.72	0.89	1.06
10	15.33	2.13	1.37	0.71	0.84
15	14.46	2.34	1.50	0.71	0.86
20	14.89	2.35	1.37	0.64	0.77

**Table 5:** Press vulcanization times for SBR compounds.

Filler % (Rice Straw/Cellulose)	Rice Straw Pressing Time (min)	Cellulose Pressing Time (min)
0	4.0	4.0
5	3.5	3.0
10	3.0	2.5
15	2.5	2.5
20	2.5	2.5

**Table 6:** Mechanical properties of SBR compounds with rice straw.

Test	0%	5%	10%	15%	20%	Recommendation
Hardness (Shore A)	56.8 ± 0.8	60.6 ± 0.6	63.6 ± 0.6	65.4 ± 0.6	67.4 ± 0.6	N/A *
Density (g/cm^3^)	1.11 ± 0.01	1.10 ± 0.01	1.11 ± 0.01	1.14 ± 0.01	1.12 ± 0.01	N/A *
Abrasion resistance (mm^3^)	157.3 ± 0.6	198.3 ± 2.1	220.3 ± 4.0	254.0 ± 13.5	294.3 ± 4.0	≤250
Tensile strength (MPa)	9.6 ± 0.7	9.9 ± 0.9	10.4 ± 0.8	10.2 ± 0.8	7.9 ± 1.0	≥8
Elongation (%)	476.9 ± 58.4	502.8 ± 48.1	555.6 ± 26.9	444.4 ± 59.1	490.7 ± 90.7	≥400
Tear resistance (N/mm)	11.0 ± 0.3	12.2 ± 0.3	19.6 ± 1.6	17.2 ± 0.6	21.3 ± 0.5	≥8

* Not applicable.

**Table 7:** Mechanical properties of SBR compounds with cellulose.

Test	0%	5%	10%	15%	20%	Recommendation
Hardness (Shore A)	56.8 ± 0.8	61.0 ± 0.7	62.2 ± 0.5	63.0 ± 0.0	61.8 ± 0.8	N/A *
Density (g/cm^3^)	1.11 ± 0.01	1.10 ± 0.01	1.12 ± 0.01	1.14 ± 0.01	1.16 ± 0.01	N/A *
Abrasion resistance (mm^3^)	157.3 ± 0.6	184.3 ± 2.5	218.3 ± 3.1	250.7 ± 1.2	280.3 ± 3.1	≤250
Tensile strength (MPa)	9.6 ± 0.7	10.1 ± 0.8	10.3 ± 0.6	10.1 ± 0.2	8.7 ± 1.1	≥8
Elongation (%)	476.9 ± 58.4	453.0 ± 22.1	433.3 ± 26.8	478.5 ± 49.8	448.4 ± 39.2	≥400
Tear resistance (N/mm)	11.0 ± 0.3	11.7 ± 0.9	12.2 ± 2.4	13.3 ± 0.5	12.5 ± 0.5	≥8

* Not applicable.

**Table 8:** Mechanical properties of TPU compounds with rice straw.

Test	0%	5%	10%	15%	20%	Recommendation
Hardness (Shore A)	82.6 ± 0.6	85.4 ± 0.6	87.2 ± 0.5	89.2 ± 0.5	90.6 ± 0.6	N/A *
Density (g/cm^3^)	1.23 ± 0.01	1.23 ± 0.01	1.21 ± 0.01	1.22 ± 0.01	1.30 ± 0.01	N/A *
Abrasion resistance (mm^3^)	35.6 ± 4.0	90.7 ± 4.0	96.7 ± 9.0	109.0 ± 6.2	90.7 ± 4.2	≤180
Tensile strength (MPa)	30.9 ± 1.5	23.4 ± 1.5	23.9 ± 2.6	18.0 ± 4.0	18.1 ± 0.0	≥7
Elongation (%)	716.4 ± 6.4	600.9 ± 9.0	621.2 ± 47.8	538.7 ± 69.2	574.7 ± 17.9	≥400
Tear resistance (N/mm)	69.5 ± 4.6	71.7 ± 6.2	70.1 ± 11.2	69.3 ± 4.4	73.6 ± 1.2	≥8

* Not applicable.

**Table 9:** Mechanical properties of TPU compounds with cellulose.

Test	0%	5%	10%	15%	20%	Recommendation
Hardness (Shore A)	82.6 ± 0.6	84.8 ± 0.5	86.0 ± 0.7	87.4 ± 0.6	88.0 ± 0.1	N/A *
Density (g/cm^3^)	1.23 ± 0.01	1.22 ± 0.01	1.24 ± 0.01	1.27 ± 0.01	1.26 ± 0.01	N/A *
Abrasion resistance (mm^3^)	35.7 ± 4.0	119.3 ± 8.0	135.3 ± 12.4	164.7 ± 35.0	267.0 ± 35.8	≤180
Tensile strength (MPa)	30.9 ± 1.5	23.6 ± 3.1	22.2 ± 1.3	19.6 ± 2.4	10.6 ± 1.1	≥7
Elongation (%)	716.4 ± 6.4	636.8 ± 69.1	661.0 ± 29.2	634.9 ± 70.7	479.4 ± 46.2	≥400
Tear resistance (N/mm)	69.5 ± 4.6	61.9 ± 9.5	61.6 ± 12.5	54.1 ± 2.5	39.8 ± 0.0	≥8

* Not applicable.

**Table 10:** DTG peak data for TPU composites with cellulose (C) and rice straw (RS), showing the percentage of mass loss at each degradation peak.

Filler Amount	Mass Loss Percentage (%)			
	Peak 1	Peak 2	Peak 3	Peak 4
0%	0.30	-	21.99	77.71
5% C	0.18	-	68.89	30.93
10% C	0.02	-	68.19	31.80
15% C	0.29	-	67.11	32.60
20% C	0.41	-	69.65	29.95
5% RS	0.33	15.98	52.26	31.44
10% RS	0.04	17.76	53.75	28.45
15% RS	0.31	20.81	54.44	24.44
20% RS	0.76	24.56	54.95	19.73

**Table 11:** DTG peak data for SBR composites with cellulose (C) and rice straw (RS), showing the percentage of mass loss at each degradation peak.

Filler Amount	Mass Loss Percentage (%)			
	Peak 1	Peak 2	Peak 3	Peak 4
0%	0.36	-	7.25	92.40
5% C	0.18	-	10.91	88.91
10% C	0.05	-	13.80	86.15
15% C	0.07	-	17.00	82.93
20% C	0.00	-	20.76	79.24
5% RS	0.42	5.25	6.13	88.20
10% RS	0.13	7.06	7.65	85.16
15% RS	0.26	7.85	8.80	83.09
20% RS	0.48	10.09	10.16	79.27

**Table 12:** DSC data for TPU composites with cellulose (C) and rice straw (RS).

Filler Amount	T_g_ (°)	T_m1_ (°)	T_m2_ (°)	T_m3_ (°)	T_m4_ (°)	ΔH_mT_ (J/g)
0%	−38.03	159.78	173.78	-	-	−7.45
5% C	−37.79	160.07	172.57	199.07	-	−3.39
10% C	−37.80	157.72	172.38	194.21	-	−2.69
15% C	−38.09	155.76	171.77	198.27	-	−2.99
20% C	−34.07	159.08	193.39	-	-	−10.15
5% RS	−35.01	162.39	176.39	190.38	201.05	−6.80
10% RS	−35.72	162.41	175.91	189.07	201.74	−7.07
15% RS	−35.34	162.92	175.75	201.42	-	−7.14
20% RS	−36.13	161.09	175.09	189.10	201.76	−9.36

**Table 13:** DSC data for SBR composites with cellulose (C) and rice straw (RS).

Filler Amount	T_g_ (°)	T_c_ (°)	ΔH_c_ (J/g)	T_m1_ (°)	T_m2_ (°)	ΔH_mT_ (J/g)
0%	−48.85	16.14	0.66	65.79	94.27	−2.13
5% C	−48.99	-	-	85.82	111.16	−2.10
10% C	−47.69	-	-	77.01	113.84	−1.32
15% C	−48.71	-	-	107.12	-	−1.20
20% C	−48.41	-	-	100.43	-	−2.40
5% RS	−48.27	23.94	1.67	92.38	-	−4.25
10% RS	−49.23	-	-	93.11	-	−2.32
15% RS	−49.33	-	-	92.45	-	−2.19
20% RS	−48.99	-	-	91.79		−1.87

**Table 14:** The *p*-value results obtained from the ANOVA for the different mechanical properties studied. The *p*-values under 0.05 are shown in red.

Source	Abrasion R. (*p*-Value)	Tensile Strength (*p*-Value)	Elongation (*p*-Value)	Tear R. (*p*-Value)
A: Material	0.0000	0.0000	0.0001	0.0000
B: Filler	0.1102	0.5125	0.6684	0.0166
C: Filler quantity	0.0012	0.0010	0.1519	0.9386
AC interaction	-	0.0023	-	-

## Data Availability

The data presented in this study are available upon request from the corresponding author.
